# Heavy metal induced resistance to herbivore of invasive plant: implications from inter- and intraspecific comparisons

**DOI:** 10.3389/fpls.2023.1222867

**Published:** 2023-08-15

**Authors:** Yue Zhou, Chao Chen, Yuntao Xiong, Feng Xiao, Yi Wang

**Affiliations:** Yunnan Key Laboratory of Plant Reproductive Adaptation and Evolutionary Ecology and Centre for Invasion Biology, Institute of Biodiversity, School of Ecology and Environmental Science, Yunnan University, Kunming, China

**Keywords:** herbivore, heavy metal pollution, invasive plant, resistance, secondary metabolites

## Abstract

**Introduction:**

Heavy metals can affect the content of secondary metabolites in plants, which are one of the important defenses of plants against herbivores. However, studies on the effects of heavy metals on secondary metabolites of invasive plants are scarce. *Phytolacca americana* is an invasive plant in China, which can hyperaccumulate the heavy metal Mn.

**Methods:**

This study used two Mn treatments (control and treatment group) and four species from Phytolacca (including the native and introduced populations of *P. americana*, its native and exotic congeners in China) to investigate the impact of heavy metal Mn on the invasive ability of *P. americana*.

**Results:**

The results show that heavy metal Mn can enhance the inhibitory effect of the introduced populations of *P. americana* on the growth of herbivore (the weight of herbivore has decreased by 66%), and altered the feeding preferences of herbivore. We also found that heavy metal Mn can significantly increase the content of quantitative resistance in the leaves of the introduced populations of *P. americana* and is higher than its native populations, native and exotic congeners. In addition, heavy metal Mn caused the quantitative resistance of the exotic congener significantly higher than that of the native congeners.

**Discussion:**

In summary, the heavy metal Mn can increase the content of secondary metabolites in leaves to enhance the interspecific competitive advantage of *P. americana* and promote its invasion, and also increase the invasion risk of exotic species.

## Graphical Abstract

Heavy metal Mn can enhance the herbivory resistance of *P. americana* leaves by enhancing their quantitative resistance, and even change the feeding preferences of herbivory, ultimately promoting invasion. The plus sign represents an increase, while the minus sign represents a decrease. "+" indicates *P* < 0.05, "++" indicates *P* < 0.01, "+++" indicates *P* < 0.001. "- -" indicates *P* < 0.01, "ns" indicates *P* > 0.05.

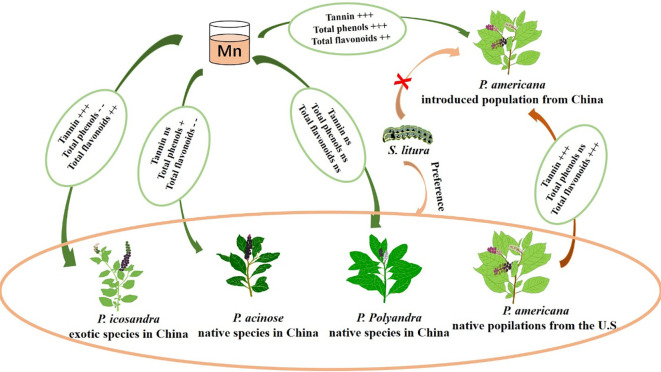

## 1 Introduction

Biological invasions not only seriously threaten and damage ecosystems and biodiversity, but also have cascading effects on the economy and pose a threat to human health (Elton, 1958; Pysek et al., 2012; Paini et al., 2016). Heavy metals are natural components of soils, but their environmental concentrations are increasing as a result of human activities such as mining and metallurgy (Pan et al., 2015; Gupta and Joia, 2016; Ashraf et al., 2019). Research has shown that some invasive plants such as *Alternanthera philoxeroides* and *Solidago canadensis* (Yuan et al., 2016; Fu et al., 2017), can tolerate high concentrations of heavy metals (Prabakaran et al., 2019), *Celosia argentea* and *Phytolacca americana* can even hyperaccumulate heavy metals in their tissues (Peng et al., 2008; Fu et al., 2011). Therefore, understanding the effects of heavy metals on invasive plants is of great significance to better analyses their invasion mechanisms and predict future expansion.

In recent years, with the complexity and diversification of environmental problems, scholars have linked global change factors such as heavy metals with biological invasion (Smith et al., 2000; Dai et al., 2020). Long period exposure to serious cadmium pollution benefits an invasive plant (*Alternanthera philoxeroides*) competing with its native congener (*Alternanthera sessilis*), the number of *A. sessilis* is inhibited, and the number of *A. philoxeroides* increases in the high cadmium levels (Wang et al., 2021b). Moreover, the Elemental Defense Hypothesis suggests that hyperaccumulated metals provide defense to plant tissues against herbivores and/or pathogens (Boyd and Martens, 1994; Boyd and Martens, 1998). Enrichment of Cd in *Eupatorium adenophora* leaves significantly inhibited the growth of pathogenic fungi (Dai et al., 2020). Selenium accumulation in *Brassica juncea* was shown to have protective effects against *Fusarium* and *Alternaria* fungal pathogens (Hanson et al., 2003). However, the effects of heavy metals on the resistance of invasive plants to herbivores are relatively scarce.

Secondary metabolites are the result of plant adaptations to the ecological environment and play an important role in protecting plants from pathogens and herbivores (Bennett and Wallsgrove, 1994; Yang et al., 2018). The secondary metabolites that are related to insect resistance are mainly alkaloids, terpenoids and phenols, which can be divided into quantitative defense and qualitative defense (Rhodes and Cates, 1976; Zhang and Liu, 2003). Quantitative defenses such as tannins, total phenols, total flavones, lignin, and cellulose (Feeny, 1975; Smilanich et al., 2016) mainly combat generalist insects, often with low synthetic cost and low content in plants, and provide protection by destroying the nervous system function of herbivores. Alternatively, qualitative defenses such as saponins and alkaloids (Feeny, 1975; Smilanich et al., 2016), are effective against all insects, and are usually associated with high synthetic costs and contents, and provide protection by reducing the growth rate of herbivores by reducing leaf tissue.

In fact, heavy metals have detrimental effects on germination, growth, nutrient uptake, as well as primary and secondary metabolism, affecting nearly all stages of the plants’ life cycle (Dalcorso et al., 2013; Shahid et al., 2017; Dubey et al., 2018). Heavy metals can affect the content of various enzymes in the secondary metabolic pathway, thus affecting the composition and content of secondary metabolites (Maksymiec et al., 2005; Foroughi et al., 2014). Studies have found that plants growing under heavy metal stress will increase the content of phenolic substances (Santiago et al., 2000). In addition, heavy metal Cd stress can lead to the production of flavonoids, tannins, phenolic acids and other secondary metabolites in *Thlaspi caerulescens*, which can interfere with or inhibit the growth, development and reproduction of insects (Llugany et al., 2013). However, to date, studies examining the effect of heavy metals on secondary metabolites of invasive plants are still lacking. Therefore, the effect of heavy metals on the resistance of invasive plants to herbivores deserves more attention.

The Enemy Release Hypothesis (ERH) states that invasive plants introduced into a new habitat are only threatened by generalist insects, thus they escape the pressure of specialized predators and successfully invade (Manea et al., 2019). Previous studies have compared the insect resistance of invasive plants to its native populations and native species from invasion site. For example, the alkaloid content of the invasive plant *Senecio jacobaea* was higher than that of ancestral populations (Siemann and Rogers, 2001) and the total leaf terpene contents of exotic plant species in Hawaii were 135% higher than that in ancestral populations (Penuelas et al., 2010). These differences may explain invasive species increased resistance to generalist herbivores compared to ancestral populations. Additionally, invasive populations of *Acacia longifolia* exhibit more biomass and terpenes along with less phenolics than their native species (Blossey and Notzold, 1995). Similarly, the invasive populations of *Sapium sebifera* grew and reproduced faster and displayed stronger stress resistance but lower chemical resistance to insects compared with the native species (Leimu et al., 2012), which explained their lower resistance to specialist herbivores and increased allocation of resources to reproduction compared to native species.

*Phytolacca americana* is an herbaceous perennial plant native to northeastern United States, which was introduced to China in 1935 and has invaded in more than 20 provinces since it was introduced (Xu et al., 2012; Ma, 2013). The entire plant of *P. americana* is poisonous, the root and fruit being the most toxic, meaning poisoning accidents can occur if *P*. *americana* were to be taken in inappropriately (Kim et al., 2005), and has been listed as invasive species in China’s natural system. *Phytolacca acinosa* and *Phytolacca polyandra* are native species in China. Among them, *P. acinosa* is a traditional Chinese medicinal plant. *Phytolacca icosandra* is an exotic species to China, native to northern Mexico and currently distributed in Kunming and Guangzhou, China, which is a kind of plant with high risk of invasion because of its strong tolerance (Xi et al., 2021). Research has found that the introduced populations of *P. americana* share the same ecological niche and have seriously threatened the survival of *P. acinosa*, an important local medicinal resource in China (Xiao et al., 2019; Liu et al., 2022). Therefore, in-depth analysis of the invasion mechanism of *P. americana* is very essential to protect *P. acinosa*. Field investigation has found that Phytolacca americana was less harmed by herbivores in the field (Xiao et al., 2019). Moreover, *P. americana* is often distributed in heavy metal Mn polluted areas, and can even hyperaccumulate heavy metal Mn in the plant tissues (Bo et al., 2005; Peng et al., 2006; Peng et al., 2008; Gao et al., 2013). Further, studies have shown that the content of secondary metabolites of *P. americana* is higher than that of other species of the Phytolacca family (Kim et al., 2005).

Thus, we proposed that: (1) Whether heavy metal Mn differentially affects herbivore resistance of *P. americana* (the native populations from the U.S. and the introduced populations from China) and its native and exotic congeners from China? (2) Whether heavy metal Mn differently affects the content of secondary metabolites in leaves of *P. americana* (the native populations from the U.S. and the introduced populations from China) and it is the native and the exotic congeners from China?

## 2 Materials and methods

### 2.1 Plants materials

Our experiment used four species from the Phytolaecaceae family: *P*. *americana* (including introduced populations from China and native populations from the U.S.), *P. acinose* (native species from China), *P. polyandra* (native species from China), *P. icosandra* (exotic species in China) (**Figure S1**). In July 2020, the mature fruits of these species were collected randomly from 15 plants. Specifically, *P*. *americana* was collected in Kunming, Yunnan (24°49′ N, 102°52′ E) and The U.S. (42°25′ N, 83°67′ W), *P. acinose* was collected in Qujing, Yunnan (25°26′ N, 104°19′ E), *P. polyandra* was collected in Diqing, Yunnan (27°35′ N, 99°09′ E), and *P. icosandra* was collected in Kunming, Yunnan (25°07′ N, 102°44′ E), China. The sampling sites were all characterized by mild human disturbance, with the distance between the sampled species being at least 2 kilometers. The experiment was conducted in a greenhouse at Yunnan University (102°52′N, 24°50′E), Kunming, China. After removing the pulp, the seeds were obtained.

Seed dormancy was broken using concentrated sulfuric acid, after which the seeds were buried under the surface of the soil and given enough water to allow for germination (Liu et al., 2020). After 24 days, seedings with 2 - 5 cotyledons were selected and transferred to plastic pots 19cm in diameter and 16.5 cm in height. All plants were randomly distributed. After two months, seedlings were randomly separated into two Mn treatment groups. The CK group was a control group, while the Mn group received an added 100 mL 10000 μM MnCl_2_·4H_2_O. Plants were cultivated for a further 75 days prior to use in experiments (5 plants × 2 treatments × 30 replicates = 300 plants). During the experimental period, the average temperature of the greenhouse was 30 °C during the day and 18 °C at night, and the relative humidity was 50 - 80%. Soil was purchased from Jiangsu Peilei Technology Development Co., Ltd. in Zhenjiang of Jiangsu, China with the following physical and chemical characteristics: 20% organic matter content, pH value 6.89, total N 8.73 g·kg^−1^, total P 2.23 g·kg^−1^, and organic carbon 85.69 g·kg^−1^.

### 2.2 Test insects

Through field observation experiments, we found that the main herbivorous insect feeding on *P. americana* was *Spodoptera litura* in China, which is a member of the Noctuidae family and is a widespread agricultural pest. *S. litura* is a generalist defoliator that is known to feed on plants in the Phytolacca family plant in the field. *S. litura* is characterized by a short generation time (about 22 ± 3d from egg to adult). To start a laboratory colony for experimentation, eggs were purchased from Ke Yun Biocontrol Company (Jiaozuo, Hennan province, China). For this study, two instars of *S. litura* larval were used. After hatching, the larvae were fed an artificial diet in an illumination incubator (12 h at 27°C with light and 12 h at 27°C without light) for later use.

### 2.3 No-choice feeding experiment

We conducted the no-choice feeding experiment of herbivores to assess the effect of heavy metal Mn on the herbivore resistance of plants according to the growth and development of the herbivore. The fifth to eighth leaf from the top of each plant were collected randomly, cut into small pieces and randomly placed on moist filter paper in a petri dish (inner diameter, 9 cm). One second instar *S. litura* larva was randomly selected and placed in a petri dish, and the initial weight of each larva was recorded. Petri dishes were closed and randomly placed in an illumination incubator (12 h at 27°C with light and 12 h at 27°C without light). The leaves in the petri dish and the filter paper were replaced every 1-2 days, and the mass of each larval was recorded every three days, with a total of three records before the larval began pupating. The increase in larval mass was calculated and the weight of each pupa was recorded (5 plants × 2 treatments × 12 replicates = 120 petri dishes).

### 2.4 Choice feeding experiment

The choice feeding experiment of herbivore was used to determine whether heavy metal Mn affected the feeding preference of herbivore. We used the leaf disc test to assess the feeding preference of herbivore between the two plants (Tang and Wang, 2007). A total of eight leaf discs of four per populations were placed equidistant in the order of ABABABAB in a petri dish with moist filter paper (**Figure S2**), which were obtained through a punch with a diameter of 1.2 ± 0.03 cm. A second instar larvae of *S. litura* was randomly selected and placed in the center of each petri dish, which was starved for 5 hours in advance. After feeding for 14 hours, photos were taken, and the feeding area was calculated using image J 2014.

### 2.5 Phytochemical analysis of plant’s secondary metabolites

A phytochemical analysis was performed to measure the effect of heavy metal Mn on secondary metabolites content in plant leaves. Three pots of each combination of plants and Mn treatments (5 plants × 2 treatments = 10 combinations) were randomly selected and 5-7 leaves from the top of each plant were collected, dried for 6 days at 60 °C, and finally ground into a powder (MM400, Retsch, Laichi, Germany). The secondary metabolites we measured include: qualitative resistance compounds (including saponins and alkaloids), quantitative resistance compounds (including total phenols, total flavonoids, tannins, cellulose, and lignin), and nutrients (including starch and soluble sugar). They were measured using purchased kits (Suzhou Grace Biotechnology Co. Ltd and Suzhou Comin Biotechnology Co., Ltd) with ultraviolet spectrophotometer analysis (UV-9000S, METASH, China) (10 combinations × 3 replicates × 9 indicators = 270 samples).

### 2.6 Statistical analysis

The weight gain of *S. litura* larvae and pupa weight were analyzed with linear models (LM). The models included heavy metal Mn treatments (n = 2) and plants (n = 5) as fixed factors, with time (n = 3) as a covariate. Similar models were also used to assess the effects of heavy metal Mn treatments (n = 2) and plants (n = 5) on quantitative resistance, qualitative resistance and nutrient content in plant leaves. Least square means *post hoc* tests (LSM) were carried out to locate significant differences among plants and heavy metal Mn treatments, and *P* values were adjusted using the LSD method. A one-way ANOVA and student’s t test were used to assess the effects of heavy metal Mn on choice feeding area of *S. litura*. The Shapiro–Wilk test of the dataset’s normality and the Levene’s test of homoscedasticity were used to determine the deviations of variance from normality and homogeneity of the data prior to analysis. All statistical tests were carried out at the 5% level of significance (α = 0.05), where *P* < 0.05 is, considered significant. All values were reported as mean ± SE. All statistical analyses were performed using the R 4.1.0 (R Core Team 2021) with the ‘lme4’, ‘car’, ‘emmeans’, ‘multcomp’, ‘MASS’, ‘psych’, ‘DescTools’ and ‘dplyr’ packages, and all diagrams were drawn using Origin 2021.

## 3 Results

### 3.1 No-choice feeding experiment

**Figure 1:**
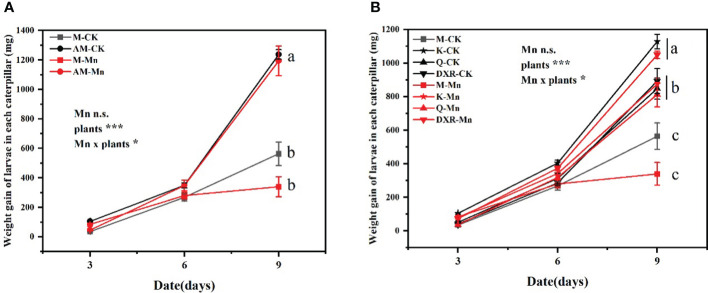
Effects of plants, heavy metal Mn and their interaction on weight gain of *S. litura* larvae. The plants include: the introduced populations of *P. americana* (from China) and the native populations of *P. americana* (from the U.S.), the native species of P. acinose and *P. polyandra* (from China), the exotic species of *P. icosandra* (in China), which was named M, AM, Q, DXR and K in the figures. **(A, B)** represent the weight gain of larvae in each caterpillar. **(A)** is compared to M and AM. **(B)** is compared to M, K, Q, and DXR. “CK” indicates that the control group does not add heavy metal Mn, and “Mn” indicates that the treatment group adds 100mL 10000mM heavy metal Mn solution to the soil. The results of liner model ANOVA are summarized in Appendix S1: **Table S1**. Data are presented as mean ± SE, n =12. Each line with the same letter is not significantly different at the 0.05 level..

The different plants had significant effects on the performance of *S. litura* (weight gain: *F*_4, 349 _= 22.9737, *P* < 0.0001; pupa fresh weight: *F*_4, 66 _= 10.3367, *P* < 0.0001; pupa dry weight: *F*_4, 66 _= 4.5665, *P* < 0.01) (**Tables S1, S2**). Specifically, the growth of *S. litura* reared with the introduced populations of *P. americana* (from China) was significantly worse than that reared with the native populations of *P. americana* (from the U. S.), *P. acinose* (native species from China), *P. polyandra* (native species from China), and *P. icosandra* (exotic species in China), regardless of whether they were treated with heavy metal Mn (**Figure 1**).

In addition, there was interactive effect between heavy metal Mn and plants on the weight gain of *S. litura* larvae (*F*_4, 349 _= 2.7561, *P* = 0.0279) (**Table S1**). The addition of heavy metal Mn will inhibit the growth of *S. litura* on the introduced populations (from China) and native populations (from the U.S) of *P. americana*. The introduced populations of *P. americana* (from China) had a stronger inhibitory effect on *S. litura* (**Figure 1A**). The fresh weight and dry weight of pupae of the introduced populations of *P. americana* (from China) were significantly lower than that of the native populations of *P. americana* (from the U.S.) after adding heavy metal Mn (*P* < 0.05, **Figure S3A**). Moreover, on the 9^th^ day, we calculated the weight gain rate of *S. litura* reared with the control leaves to weight gain rate of *S. litura* reared with heavy metal-treated leaves. Specifically, the introduced populations of *P. americana* (from China) was 1.66 times, *P. icosandra* (exotic species in China) was 1.29 times, *P. acinose* (native species from China) was 1.04 times, and *P. polyandra* (native species from China) was 0.85 times. After heavy metal Mn addition, the fresh weight of *S. litura* pupae decreased significantly in the introduced populations of *P. americana* (from China) and *P. icosandra* (exotic species in China) (*P* < 0.05, **Figure S1**).

### 3.2 Choice feeding experiment

**Figure 2:**
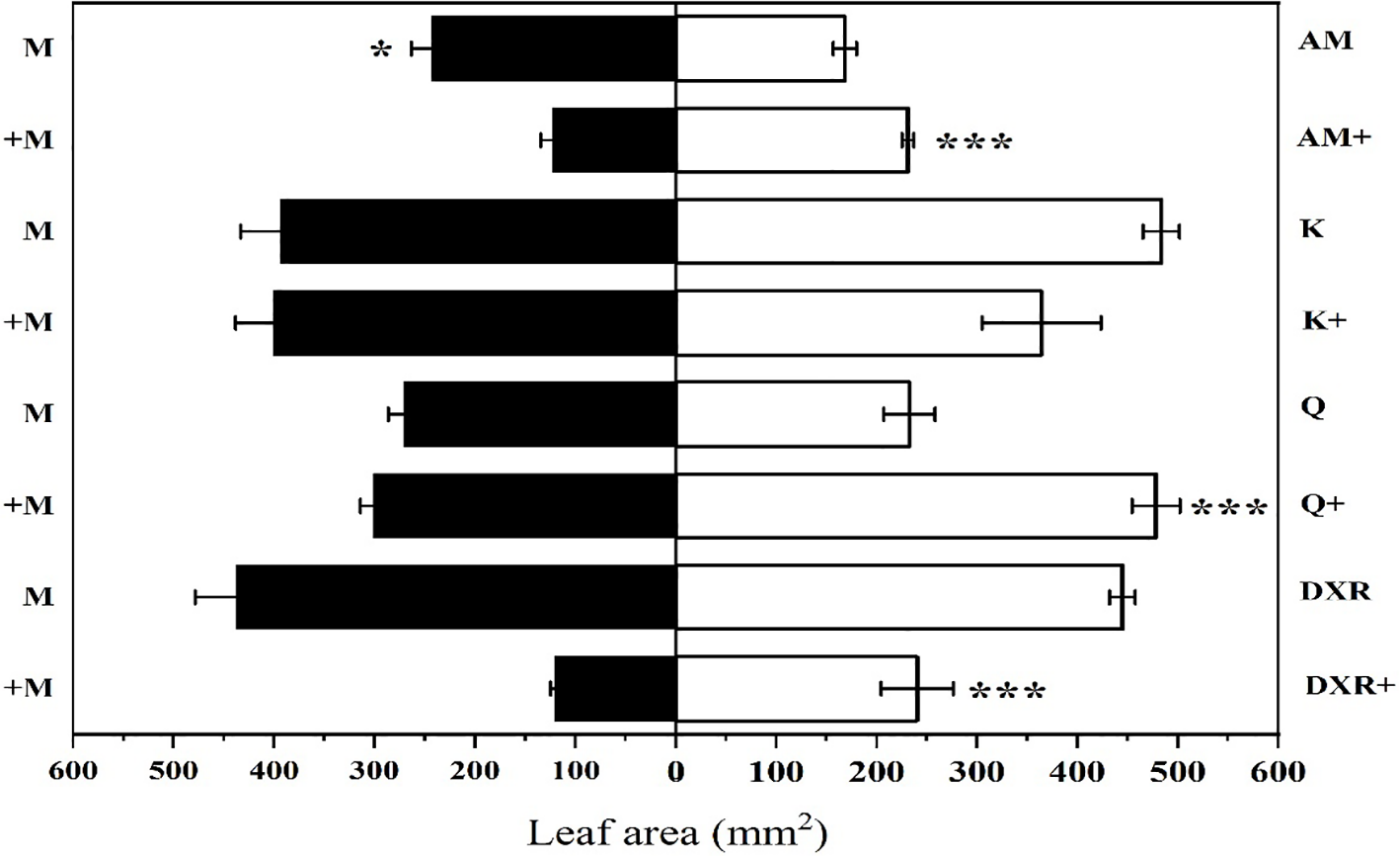
Choice feeding of *S.litura* between two plants. The plants include: the introduced populations of *P. americana* (from China) and the native populations of *P. americana* (from the U.S.), the native species of *P. acinose* and *P. polyandra* (from China), the exotic species of *P. icosandra* (in China), which was named M, AM, Q, DXR and K in the figures. “-” indicates that the control group does not add heavy metals Mn, and “+” indicates that the treatment group adds 100mL 10000μM heavy metal Mn solution to the soil. Data are shown as mean ± SE, n = 6. Asterisk are shown for significance, unmarked are not significantly different. “***”, *P* < 0.001, “*”, *P* < 0.05.

*S. litura* preferred to eat the introduced populations of *P. americana* (from China) compared with the native populations of *P. americana* (from the U.S.) in the control group (*P* < 0.05, **Figure 2**). However, *S. litura* preferred to eat the native populations of *P. americana* (from the U.S.) after adding heavy metal Mn (*P* < 0.05, **Figure 2**). In addition, *S. litura* had no obvious feeding preference for either the introduced populations of *P. americana* (from China), *P. acinose* (native species from China), *P. icosandra* (exotic species in China), or *P. polyandra* (native species from China) in the control group (*P* > 0.05), however, *S. litura* significantly preferred to feed on *P. acinose* and *P. polyandra* after adding heavy metal Mn (*P* < 0.001, **Figure 2**).

### 3.3 Phytochemical analysis of plant qualitative resistance

**Figure 3:**
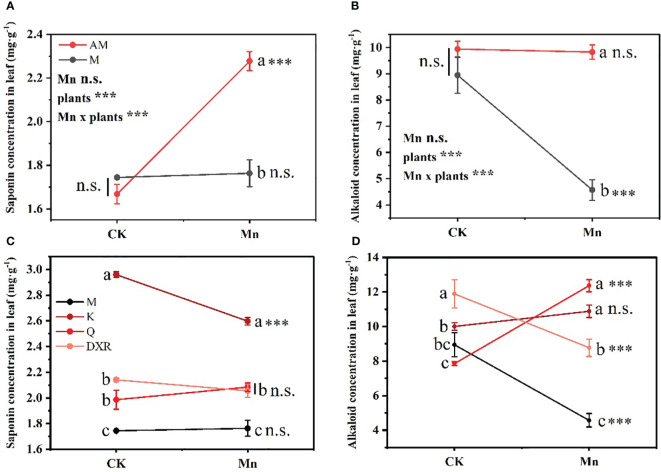
Effects of plants, heavy metal Mn and their interaction on leaf qualitative resistance of plants. Qualitative resistance compound include: Saponins **(A, C)** and Alkaloids **(B, D)**. The plants include: the introduced populations of *P. americana* (from China) and the native populations of *P. americana* (from the U.S.), the native species of *P. acinose* and *P. polyandra* (from China), the exotic species of *P. icosandra* (in China), which was named M, AM, Q, DXR and K in the figures. “CK” indicates that the control group does not add heavy metals Mn, and “Mn” indicates that the treatment group adds 100 mL 10000 μM heavy metal Mn solution to the soil. The results of liner model ANOVA are summarized in Appendix S1: **Table S3**. Data are shown as mean ± SE, n = 3. Letter codes shown represent the results of multiple comparisons between different plants under the same heavy metal Mn treatment, and asterisks show the results of multiple comparisons between two heavy metal Mn treatments under the same plants; “***”, *P* < 0.001. The same letter code and “ns” are not significantly different (*P* > 0.05).

Different plants and heavy metal Mn had interactive effects on the qualitative resistance of plant leaves (Saponin: *F*_4, 20 _= 22.7859, *P* < 0.001; Alkaloid: *F*_4, 20 _= 20.1048, *P* < 0.001) (**Table S2**). There was no significant difference in the content of qualitative resistance in the leaves of the introduced populations (from China) and the native populations (from the U.S.) of *P. americana* in the control group (**Figures 3A, B**). After adding heavy metal Mn, the qualitative resistance of the native populations of *P. americana* (from the U.S.) increased significantly, which was mainly seen in the significant increase of saponin content (*P* < 0.0001, **Figure 3A**), and the qualitative resistance of the introduced populations of *P. americana* (from the China) decreased significantly, mainly manifested in the significant decrease of alkaloid content (*P* < 0.0001, **Figure 3B**). In addition, the qualitative resistance of the introduced populations of *P. americana* (from China) was significantly lower than that of *P. icosandra* (exotic species in China), *P. acinose* (native species from China) and *P. polyandra* (native species from China) after adding heavy metal Mn (**Figures 3C, D**), including saponins and alkaloids. Moreover, heavy metal Mn addition leads to a significant decrease in the saponins in the leaves of *P. icosandra* (exotic species in China) and the alkaloid content in the leaves of *P. polyandra* (native species from China) (*P* < 0.0001, **Figures 3C, D**), and a significant increase in the alkaloid content in the leaves of *P. acinose* (native species from China) (*P* < 0.0001, **Figure 3D**).

### 3.4 Phytochemical analysis of plant quantitative resistance

**Figure 4:**
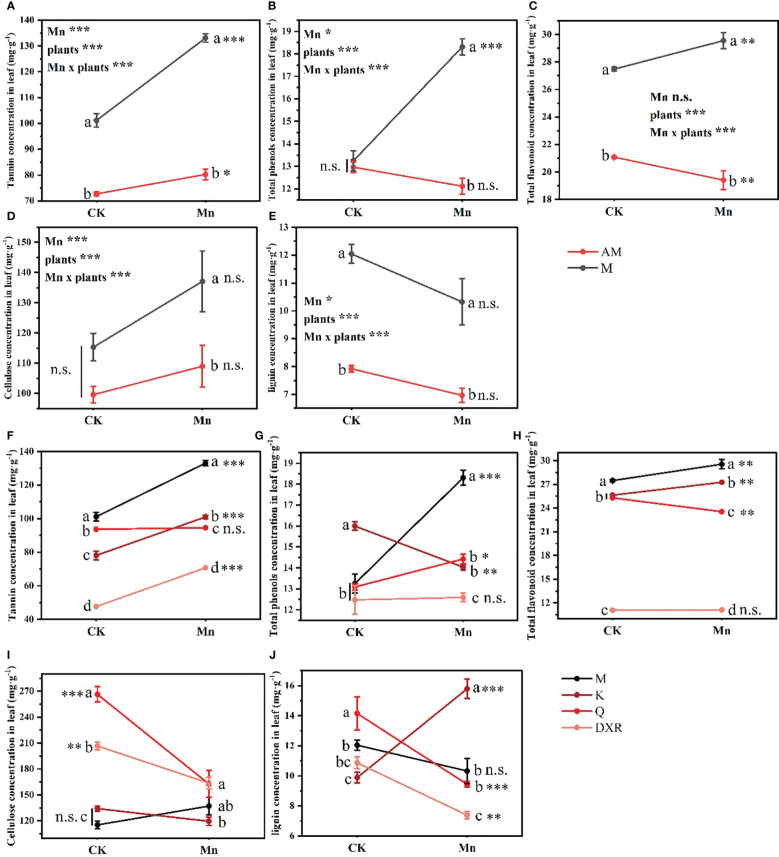
Effects of plants, heavy metal Mn and their interaction on leaf quantitative resistance of plants. Quantitative resistance include: tannin **(A, F)**, total phenols **(B, G)**, total flavonoid **(C, H)**, cellulose **(D, I)**, lignin **(E, J)**. The plants include: the introduced populations of *P. americana* (from China) and the native populations of *P. americana* (from the U.S.), the native species of *P. acinose* and *P. polyandra* (from China), the exotic species of *P. icosandra* (in China), which was named M, AM, Q, DXR and K in the figures. “CK” indicates that the control group does not add heavy metal Mn, and “Mn” indicates that the treatment group adds 100 mL 10000 μM heavy metal Mn solution to the soil. The results of liner model ANOVA are summarized in Appendix S1: **Table S3**. Data are shown as mean ± SE, n = 3. Letter codes shown represent the results of multiple comparisons between different plants under the same heavy metal Mn treatment, and asterisks show the results of multiple comparisons between two heavy metal Mn treatments under the same plants; “***”, *P* < 0.001, “**”, *P* < 0.01, “*”, *P* < 0.05. The same letter code and “ns” are not significantly different (*P* > 0.05).

Heavy metal Mn and plants interacted with the content of quantitative resistance in plant leaves (Tannin: *F*_4,20 _= 21.235, *P* < 0.001, Total phenols: *F*_4,20 _= 20.884, *P* < 0.001, Total flavonoid: *F*_4,20 _= 10.439, *P* < 0.001, Cellulose: *F*_4,20 _= 14.053, *P* < 0.001, Lignin: *F*_4,20 _= 19.777, *P* < 0.001). Notably, after heavy metal Mn addition, the quantitative resistance in the leaves of the introduced populations of *P. americana* (from China), including tannin, total phenols, total flavonoids, cellulose and lignin, were significantly higher than that of the native populations of *P. americana* (from the U.S.) (*P* < 0.05, **Figures 4A–E**). Moreover, the contents of tannins, total phenols and total flavonoids in the leaves of the introduced populations of *P. americana* (from China) increased significantly, and were significantly higher than that of *P. icosandra* (exotic species in China), *P. acinose* (native species from China) and *P. polyandra* (native species from China) after adding heavy metal Mn (**Figures 4F–H**). The content of cellulose in the leaves of introduced populations (from China) of *P. americana* was significantly lower than that of *P. acinose* (native species from China) and *P. polyandra* (native species from China) in the control group (*P* < 0.001), however, there was no significant difference in the content of cellulose after heavy metal Mn addition (**Figure 4I**). The content of lignin in the leaves of *P. acinose* (native species from China) and *P. polyandra* (native species from China) decreased significantly (*P* < 0.001) due to heavy metal Mn addition, while the introduced populations of *P. americana* (from China) had no significant change (*P* > 0.05, **Figure 4J**). The content of lignin in the leaves of *P. icosandra* (exotic species in China) increased significantly (*P* < 0.001), and was significantly higher than that of the introduced populations of *P. americana* (from China), *P. acinose* (native species from China) and *P. polyandra* (native species from China) (*P* < 0.001, **Figure 4J**).

### 3.5 Phytochemical analysis of plant’s nutrients

**Figure 5:**
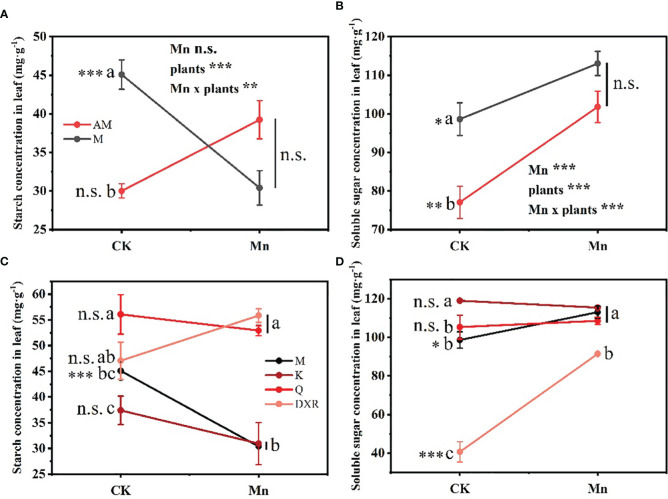
Effects of plants, heavy metal Mn and their interaction on leaf quantitative resistance of plants. Nutrient substance include: starch **(A, C)** and soluble sugar **(B, D)**. The plants include: the introduced populations of *P. americana* (from China) and the native populations of *P. americana* (from the U.S.), the native species of *P. acinose* and *P. polyandra* (from China), the exotic species of *P. icosandra* (in China), which was named M, AM, Q, DXR and K in the figures. “CK” indicates that the control group does not add heavy metals Mn, and “Mn” indicates that the treatment group adds 100 mL 10000 μM heavy metal Mn solution to the soil. The results of liner model ANOVA are summarized in Appendix S1: **Table S3**. Data are shown as mean ± SE, n = 3. Letter codes shown represent the results of multiple comparisons between different plants under the same heavy metal Mn treatment, and asterisks show the results of multiple comparisons between two heavy metal Mn treatments under the same plants; “***”, *P* < 0.001, “**”, *P* < 0.01, “*”, *P* < 0.05. The same letter code and “ns” are not significantly different (*P* > 0.05).

Heavy metal Mn and plants interacted differently with the content of nutrients in plant leaves (Starch: *F*_4, 20 _= 4.9885, *P* < 0.01, Soluble sugar: *F*_4, 20 _= 4.9885, *P* < 0.001). The contents of nutrients, including starch and soluble sugar, in the leaves of the introduced populations of *P. americana* (from China) in the control group were significantly higher than those in the native populations of *P. americana* (from the U.S.); however, there was no significant difference in nutrients after heavy metal Mn addition (**Figures 5A, B**). Moreover, after heavy metal Mn addition, the starch content in the leaves of the introduced populations of *P. americana* (from China) decreased significantly (*P* < 0.05), which was significantly lower than that of *P. acinose* (native species from China) and *P. polyandra* (native species from China) (*P* < 0.001, **Figure 5C**). The content of soluble sugar in the leaves of the introduced populations of *P. americana* (from China) and *P. polyandra* (native species from China) increased significantly after heavy metal Mn addition (*P* < 0.05, **Figure 5D**).

## 4 Discussion

This study investigated the effect of heavy metal Mn on the resistance of different species in the Phytolacca family to herbivores. We found that heavy metal Mn addition not only increased the resistance of the introduced populations of *P. americana* (from China) to herbivory, but also changed the feeding preference of herbivores. In addition, the leaf content of secondary metabolites also changed significantly after adding heavy metal Mn, which mainly manifested in the significant reduction of qualitative resistance and the significant increase of quantitative resistance, compared with the native populations of *P. americana* (from the U.S.), *P. acinose* (native species from China), *P. polyandra* (native species from China) and *P. icosandra* (exotic species in China).

### 4.1 Heavy metal Mn enhance the resistance of *P. americana* to herbivorous insects

Previous studies have found that herbivore feeding is one of the key factors affecting the successful invasion of invasive plants (Lurie et al., 2017), and heavy metal can affect plant–herbivorous interactions (Baker, 1987; Muller et al., 2004). Moreover, research has shown that heavy metals not only support the colonization of invasive plants to new environments, but also enhance their antibacterial properties (Dai et al., 2020; Wang et al., 2021a; Wang et al., 2021b). In this study, heavy metal Mn addition inhibited the development of *S. litura* larvae and decreased the weight of pupae, meanwhile, the introduced populations of *P. americana* (from China) has the strongest inhibition effect (**Figures 1**, **S3**). Other findings corroborate this, and show that heavy metals can increase plant resistance. For example, with the increase of Cd concentration, the leaf feeding damage index of *Thlaspi caerulescens* decreased significantly (Jiang et al., 2005). Additionally, selenium accumulation in *Brassica juncea* hindered herbivorous insects (Hanson et al., 2003). In the present study, we measured the content of heavy metals Mn in plant leaves. The results showed that the concentration of heavy metals in plant leaves of five plants increased significantly after heavy metal Mn addition, but this increase was in the normal range (20 - 400 μg·g^-1^) (**Figure S4**), which would not affect the growth and performance of *S. litura* (Coleman et al., 2005; Kula et al., 2014).Therefore, we believe that the increase in quantitative resistance after the addition of heavy metal Mn is an important factor in increasing plant insect resistance. Similarly, no protective effect of zinc was found in *Arabidopsis halleri*, and as such, were suspect that secondary metabolites may be involved in the observed deterrence of herbivores (Macnair, 2003).

### 4.2 Heavy metal Mn enhances the quantitative resistance of *P. americana*

The secondary metabolites produced by invasive plants help to resist herbivores and bacteria, which gives the invaders a competitive advantage during introduction (Haribal and Enwick, 1998). In the present study, heavy metal Mn addition affected the feeding preference of *S. litura*, with *S. litura* preferring the native populations of *P. americana* (from the U.S.), *P. acinose* (native species from China), *P. polyandra* (native species from China) and *P. icosandra* (exotic species in China) compared with the introduced populations of *P. americana* (from China) (**Figure 2**). This is likely due to the fact that after the heavy metal Mn addition, the quantitative resistance (including tannin, total phenols, total flavonoid) in the leaves of the introduced populations of *P. americana* (from China) significantly increased compared with other species, which is not conducive to the growth and performance of *S. litura* (**Figures 4A, C, D**). Tannin is the most abundant secondary metabolite produced by plants, and has a negative effect on the development and reproduction of herbivores by reducing digestion (Bernays, 1981; Barbehenn and Constabel, 2011). Studies have found that when *S. litura* diets contain 0.5% tannin, the need to detoxify enzymes *in vivo* increases (Huang and Li, 2018). Total phenols and total flavonoids are known to increase plant resistance to not only biological stress (e.g., herbivores) but also abiotic stress (e.g., climate) (Harborne and Williams, 2000; Treutter, 2005). When the concentration of flavonoids reaches 0.07%, *Arachis hypogaea* will have an antifeedant effect on aphids (Grayer et al., 1992). Research has shown in other species, such as *Alternanthera philoxeroides*, that the energy addition of secondary metabolites such as tannins and total phenols will enhance their ability to defend against natural enemies (Ma and Wang, 2004).

Plants can adapt to biotic and abiotic stress through the production of different types and amounts of secondary chemicals (Moreira et al., 2014). In our study, after heavy metal Mn addition, the qualitative resistance (alkaloids) in the leaves of the introduced populations of *P. americana* (from China) decreased significantly, which was significantly lower than that of the native populations (from the U.S.), and the quantitative resistance (including tannin, total phenols, total flavonoids) increased significantly, which was significantly higher than that of the native populations (from the U.S.) (**Figures 3**–**5**). No significant difference in growth between the introduced populations *Lepidium draba* and the native populations was observed, but the introduced populations did produce more of the resistant substance glucosinolate (Muller and Martens, 2005). Additionally, the resistance level of the introduced populations was also found to be higher than that of the native populations in *Chromolaena odorata* (Liao et al., 2014). These findings suggest that the EICA hypothesis does not hold for all invasive species, including *P. americana*. Moreover, we found that the starch content in the leaves of the introduced populations of *P. americana* decreased significantly after heavy metal Mn addition, which may be due to the significant increase in the quantitative resistance in the leaves of the introduced populations of *P. americana* consuming more resources. Further, heavy metal Mn addition also resulted in a significant increase in soluble sugar content in leaves of the introduced populations of *P. americana* (from China), which is consistent with previous findings that the content of soluble sugar in *Conyza Canadensis* leaves increased with the increasing concentration of heavy metal Mn (Zhang et al., 2021).

### 4.3 Defences of *P. americana* against herbivore

Notably, some studies have found that heavy metals can promote the accumulation of plant secondary metabolites, but the content of secondary metabolites decreases with the increase of heavy metal concentration or the extension of stress time (Komaraiah et al., 2002; Peng et al., 2010; Weber et al., 2010; Vakili et al., 2012). In recent years, the element defense hypothesis has been widely studied, suggesting that the element defense ability of plants will improve with the increase of heavy metals (Scheirs et al., 2006; Stolpe et al., 2017). In particular, *P. americana* is a plant that has been shown to hyperaccumulate heavy metal Mn (Peng et al., 2006; Peng et al., 2008; Gao et al., 2013). Therefore, we assumed that with the increase of heavy metal Mn concentration, the content of secondary metabolites in the leaves of the introduced populations of *P. americana* (from China) would reduce, the element defense ability would improve, and the resistance to herbivores would strengthen, however, our results suggest that this hypothesis requires further investigation. Under the strong influence of human interference, plant invasion and heavy metal pollution are common (Li et al., 2022). Our study found that the heavy metal manganese can improve the resistance and competitive ability of invasive plants of *P. americana*, providing a new perspective for the analysis of the invasion mechanism of *P. americana*. In addition, we also found that heavy metals may increase the invasion risk of the exotic species (*P. icosandra*), which provided a theoretical basis for the management measures of *P. icosandra*.

Invasive plants have formed a series of complex defense systems to deal with the feeding threat of herbivores. Our study found that the quantitative resistance in the leaves of the introduced populations of *P. americana* (from China) increased significantly and more than that of the *P. acinose* (native species from China) and *P. polyandra* (native species from China), and the qualitative resistance decreased significantly and less than that of the *P. acinose* (native species from China), *P. polyandra* (native species from China) and *P. icosandra* (exotic species from China). This finding shows that the introduced populations of *P. americana* (from China) relied on quantitative resistance, while the *P. acinose* (native species from China), *P. polyandra* (native species from China) and *P. icosandra* (exotic species from China) relied on qualitative resistance. Previous studies have found that plants have a long life and grow in patches, which is beneficial for herbivores; therefore, the defense strategies of evolutionary plants tend to reduce the use of food resources by herbivores and produce substances that inhibit the digestibility of herbivores, demonstrating quantitative defense characteristics (Feeny, 1975; Feeny, 1976; Rhodes and Cates, 1976). Therefore, the introduced populations of *P. americana* (from China) will increase quantitative resistance and promote invasion. We also found that the addition of heavy metals resulted in a significant increase in quantitative resistance (including tannin, total flavonoids and lignin) in *P. icosandra* (exotic species in China) and was significantly higher than that in the native species from China (*P. acinose* and *P. polyandra*). In addition, the feeding preference of *S. litura* did not change after the addition of heavy metals between the introduced populations of *P. americana* (from China) and *P. icosandra* (exotic species in China). Lignin resists insects by reducing the palatability of leaves (Wardle et al., 2002). These results indicated that in heavy metal polluted areas, the insect resistance of *P. icosandra* (exotic species in China) was stronger than that of the native species. Some studies have predicted that the species is likely to successfully invade China and further harm the Chinese ecosystem (Xi et al., 2021). Therefore, we should strengthen the management of *P. icosandra* (exotic species in China). Further, invasive plants can also transfer heavy metals to higher nutritional levels through the food chain, the implications of which require additional investigation in the future (Harvey et al., 2003; Heleno et al., 2009; Wang et al., 2019).

## 5 Conclusion

Our study found that heavy metal Mn remarkably increased the content of quantitative resistance (includinfg tannin, total phenols and total flavonoids) in the leaves of the introduced populations of *P. americana* (from China). This will lead to poor growth and performance of herbivores, and even affect the feeding preference of herbivores. Simply, heavy metal Mn induced resistance to herbivore of the introduced populations of *P. americana* (from China). In addition, heavy metal Mn can also enhance the resistance of exotic species in China (*P. icosandra*) (including tannin, total flavonoids and lignin), thus enhancing their competitiveness with native species from China, leading to increased risk of invasion. These results provide an important basis for the mechanisms involved in the successful invasion of *P. americana* into heavy metal Mn contaminated soil and implications for invasive plants management under environmental pollution in China.

## Data availability statement

The raw data supporting the conclusions of this article will be made available by the authors, without undue reservation.

## Ethics statement

Ethical review and approval were not required for the study on animals in accordance with the local legislation and institutional requirements.

## Author contributions

YW designed the experiments. YZ, CC, YTX and FX performed the experiments. YZ analyzed the data, drew the figures and written the paper. All authors contributed to the article and approved the submitted version.
