# Supplementary material for: Heavy metal induced resistance to herbivore of invasive plant: implications from inter- and intraspecific comparisons
Source: Front Plant Sci. 2023 Aug 15;14:1222867. doi: 10.3389/fpls.2023.1222867 (PMC10464952; doi:10.3389/fpls.2023.1222867)
Supplement: Supplementary file 1 [file DataSheet_1.docx]

**Supporting Information**

**Heavy metal induced resistance to herbivore of invasive plant: Implications from inter- and intraspecific comparisons**

Yue Zhou^1^, Chao Chen^1^, Yuntao Xiong^1^, Feng Xiao^1^, Yi Wang^1*^

^1^ Yunnan Key Laboratory of Plant Reproductive Adaptation and Evolutionary Ecology and Centre for Invasion Biology, Institute of Biodiversity, School of Ecology and Environmental Science, Yunnan University, Kunming 650504, China;

*Correspondence author: Yi Wang, E-mail: [yiwang@ynu.edu.cn,](mailto:yiwang@ynu.edu.cn,) Tel & Fax: 0086-0871-65031317

Appendix S1: Table S1. Effects of heavy metal Mn and different plants on the weight of *S. litura* larvae in non-choice feeding experiment. Time as a covariate.

|  | | Larvae weight | |
| --- | --- | --- | --- |
| Effect | df | F | *P* |
| Time | 1,349 | 1115.9792 | **<0.0001** |
| plants | 4,349 | 22.9737 | **<0.0001** |
| Treatment | 1,349 | 1.5322 | 0.2166 |
| Plants: Treatment | 4,349 | 2.7561 | **0.0279** |

|  |  | Pupa fresh weight | | Pupa dry weight | | |
| --- | --- | --- | --- | --- | --- | --- |
| Effect | df | F | *P* | F | *P* |  |
| Plants | 4,66 | 10.3367 | **<0.0001** | 4.5665 | **0.0026** |  |
| Treatment | 1,66 | 3.2063 | 0.0779 | 3.5115 | 0.0654 |  |
| Plants: Treatment | 4,66 | 2.1492 | 0.0845 | 0.9441 | 0.4442 |  |

Appendix S1: Table S2. Effects of heavy metal Mn and different plants on pupal weight of *S. litura* larvae in non-choice feeding experiment.

|  | | Lignin | | Cellulose | | Saponin | |
| --- | --- | --- | --- | --- | --- | --- | --- |
| Effect | df | F | *P* | F | *P* | F | *P* |
| Plants | 4,20 | 21.8522 | **<0.0001** | 48.2930 | **<0.0001** | 107.7993 | **<0.0001** |
| Treatment | 1,20 | 5.7195 | **0.0267** | 19.080 | **<0.0001** | 2.8051 | 0.1095 |
| Plants: Treatment | 4,20 | 19.7774 | **<0.0001** | 14.053 | **<0.0001** | 22.7859 | **<0.0001** |

Appendix S1: Table S3. Effects of heavy metal Mn and different plants on the content of resistant substances and nutrients in plant leaves.

|  | | Total Flavonoid | | Total Phenols | | Tannin | |
| --- | --- | --- | --- | --- | --- | --- | --- |
| Effect | df | F | *P* | F | *P* | F | *P* |
| Plants | 4,20 | 621.6374 | **<0.0001** | 24.2990 | **<0.0001** | 243.335 | **<0.0001** |
| Treatment | 1,20 | 0.0675 | 0.7976 | 7.8336 | **0.0112** | 196.782 | **<0.0001** |
| Plants: Treatment | 4,20 | 10.4395 | **<0.0001** | 20.8841 | **<0.0001** | 21.235 | **<0.0001** |

|  | | Alkaloid | | Starch | | Soluble sugar | |
| --- | --- | --- | --- | --- | --- | --- | --- |
| Effect | df | F | *P* | F | *P* | F | *P* |
| Plants | 4,20 | 15.7793 | **<0.0001** | 17.8956 | **<0.0001** | 38.880 | **<0.0001** |
| Treatment | 1,20 | 1.6205 | 0.2176 | 0.3745 | **0.5475** | 39.1650 | **<0.0001** |
| Plants: Treatment | 4,20 | 20.1048 | **<0.0001** | 4.9885 | **0.0059** | 11.0750 | **<0.0001** |

Appendix S1:
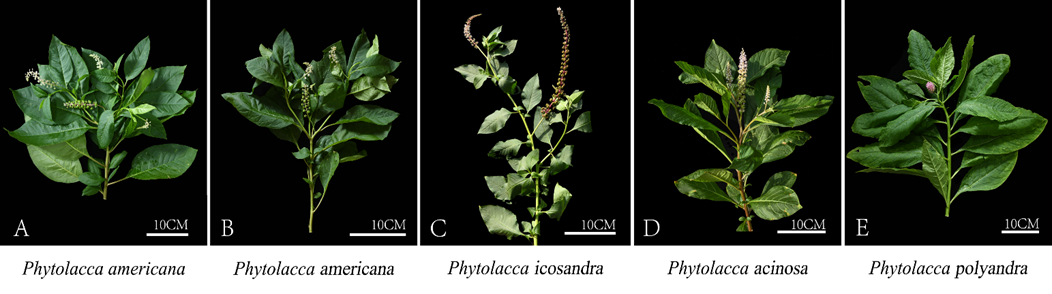
 Fig. S1. Morphological maps of four species of Phytolacca. A is the introduced populations of *Phytolacca americana* from China, B is the native populations of *Phytolacca americana* from the U.S., C is the exotic species of *Phytolacca icosandra* from China, D and E are the native species of *Phytolacca acinose* and *Phytolacca polyandra* from China. Morphological description of the four species of Phytolacca: *P. americana*, the racemes are slender and longer than the leaves and sparse, carpel connate, drooping infructescence, tepals are light red; *P. icosandra*, the racemes are usually tight and longer than leaves, carpel connate, erect to drooping infructescence, tepals are pale yellowish green; *P. acinose*, the racemes are usually shorter than the leaves and tight, carpel distinct, erect infructescence, tepals are white-green; *P. polyandra*, the racemes are usually shorter than the leaves and tight, carpel connate, erect to drooping infructescence, tepals are pink.


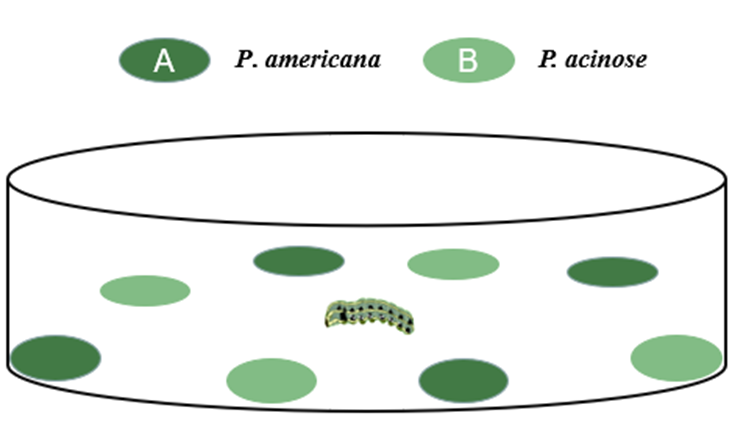


Appendix S1: Fig. S2. Schematic diagram of choice feeding experiment


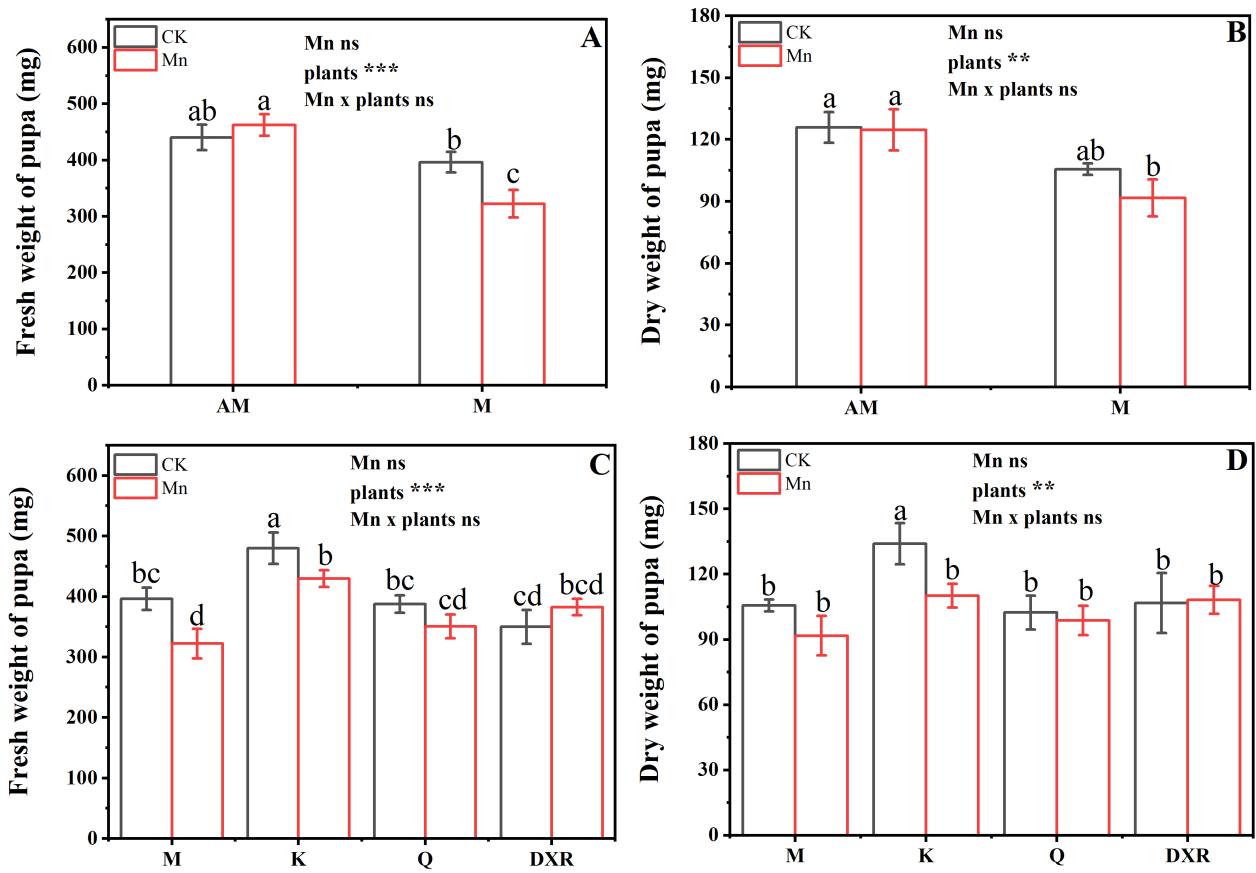


Appendix S1: Fig. S3. Effects of plants, heavy metal Mn and their interaction on pupa weight (including: pupa fresh weight and pupa dry weight). Data are shown as mean ± SE, n = 6. The plants include: the introduced populations of *P*. *americana* (from China) and the native populations of *P*. *americana* (from the U.S.), the native species of *P. acinose* and *P. polyandra* (from China), the exotic species of *P. icosandra* (from China), which was named M, AM, Q, DXR and K in the figures. "CK" indicates that the control group does not add heavy metals Mn, and "Mn" indicates that the treatment group adds 100 mL 10000 μM heavy metal Mn solution to the soil. Letter codes shown represent the results of multiple comparisons. The same letter code is not significantly different (*P* > 0.05)


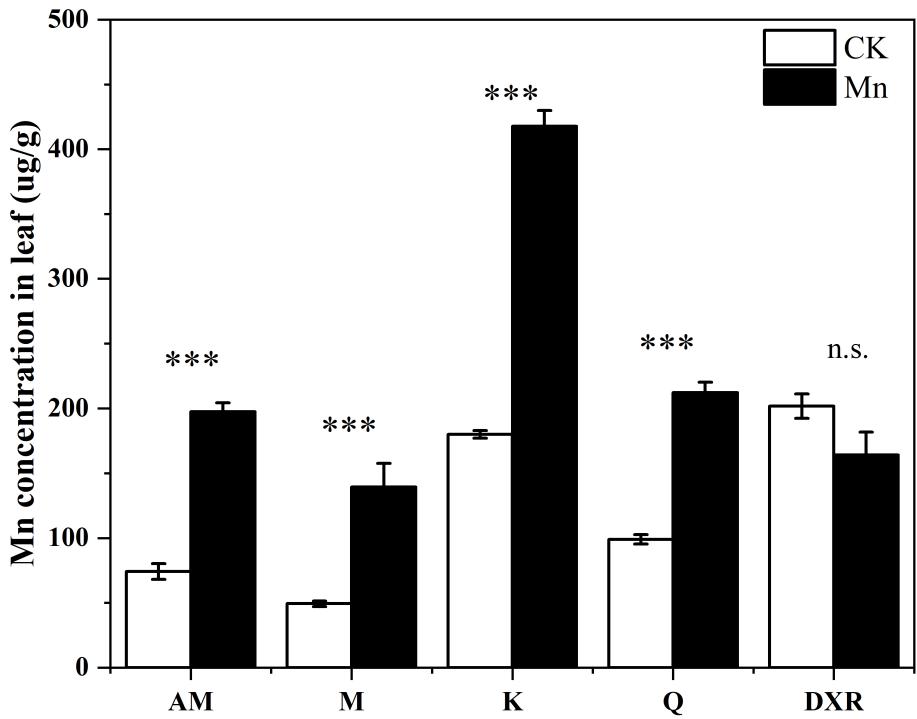


Appendix S1: Fig. S4. Mn concentration in plant leaves. Data are shown as mean ± SE, n =3. The plants include: the introduced populations of *P*. *americana* (from China) and the native populations of *P*. *americana* (from the U.S.), the native species of *P. acinose* and *P. polyandra* (from China), the exotic species of *P. icosandra* (from China), which was named M, AM, Q, DXR and K in the figures. "CK" indicates that the control group does not add heavy metals Mn, and "Mn" indicates that the treatment group adds 100 mL 10000 μM heavy metal Mn solution to the soil. Asterisks indicate significant differences between differences treatments of one species. "*", significance, "***", *P* < 0.001, "**", *P* < 0.01, "*", *P* < 0.05. The "ns" is not significantly different (*P* > 0.05).
